# Microstructural white‐matter abnormalities and their relationship with cognitive dysfunction in obsessive–compulsive disorder

**DOI:** 10.1002/brb3.442

**Published:** 2016-02-05

**Authors:** Paola Magioncalda, Matteo Martino, Benjamin A. Ely, Matilde Inglese, Emily R. Stern

**Affiliations:** ^1^ Department of Neuroscience, Rehabilitation, Ophthalmology, Genetics and Maternal and Child HealthSection of PsychiatryUniversity of GenoaGenoaItaly; ^2^ Department of NeuroscienceIcahn School of Medicine at Mount SinaiNew York CityNew York; ^3^ Department of NeurologyIcahn School of Medicine at Mount SinaiNew York CityNew York; ^4^ Department of PsychiatryIcahn School of Medicine at Mount SinaiNew York CityNew York

**Keywords:** Cognitive dysfunction, diffusion tensor imaging, obsessive–compulsive disorder, tract‐based spatial statistics, white matter

## Abstract

**Background:**

In recent years, diffusion tensor imaging (DTI) studies have detected subtle microstructural abnormalities of white matter (WM) in obsessive–compulsive disorder (OCD). However, findings have been inconsistent, and it is unclear whether WM abnormalities are related to cognitive processes. The aim of this study was to explore the relationship of WM alterations with cognitive variables in OCD in order to investigate the structural correlates of behaviorally relevant features of the disorder.

**Methods:**

We compared DTI‐derived fractional anisotropy (FA), mean diffusivity (MD), and radial diffusivity (RD) measures between OCD patients (*n* = 16) and healthy controls (*n* = 18) using a whole‐brain tract‐based spatial statistics (TBSS) approach. We also explored the correlations of WM alterations with clinical and cognitive variables.

**Results:**

Patients with OCD demonstrated increases in MD in the bilateral posterior corona radiata; left anterior corona radiata; bilateral superior longitudinal fasciculus; genu, body, and splenium of the corpus callosum; and left posterior limb of the internal capsule. An increase in RD values was also found in some of the same tracts (right posterior corona radiata, right superior longitudinal fasciculus, left anterior corona radiata, and corpus callosum). Furthermore, increased MD value in the internal capsule was correlated with the percentage of errors made during a target detection task, which was greater in the OCD group overall.

**Conclusions:**

These findings indicate that OCD patients show greater diffusivity in several white‐matter regions. The correlation between cognitive performance and diffusivity in the internal capsule suggests that microstructural WM alternations may have functional consequences for the disorder.

## Introduction

Obsessive–compulsive disorder (OCD) is a chronic and disabling neuropsychiatric disorder characterized by the presence of obsessions (i.e., recurrent, persistent, and intrusive thoughts or images that cause anxiety) and/or compulsions (i.e., repetitive behaviors aimed at reducing anxiety) (Koch et al. 2014). In recent years, investigation into the neurobiological underpinnings of OCD has made substantial progress. In particular, functional neuroimaging studies have identified altered activity in the fronto‐striato‐thalamo‐cortical circuitry (Menzies et al. 2008a) and widespread regions of the cortex (Stern and Taylor 2014). Volumetric studies are partly in line with these findings, with meta‐analyses revealing decreased gray‐matter volume in the anterior cingulate gyri (Radua and Mataix‐Cols 2009; Radua et al. 2010) and increased gray‐matter volume in the basal ganglia (putamen and caudate) and thalamus (Radua and Mataix‐Cols 2009; Radua et al. 2010; Eng et al. 2015). Furthermore, diffusion tensor imaging (DTI) studies have detected white‐matter (WM) alterations in OCD in tracts connecting many cortical and subcortical regions implicated in the disorder (Piras et al. 2013; Koch et al. 2014; Radua et al. 2014; Eng et al. 2015). DTI is a MRI technique that measures the random movement of water molecules in the brain, providing information about WM microstructure in vivo (Alger 2012). Distinct DTI parameters can be used to characterize diffusion and, indirectly, fiber tract microstructure. Two important measures of diffusion are fractional anisotropy (FA), which measures the anisotropic diffusion of water molecules, and mean diffusivity (MD), which measures the average diffusion across the *x*,* y*, and *z* directions. Additionally, radial diffusivity (RD) describes the diffusion perpendicular to axons; this measure is thought to be particularly sensitive to demyelination (Song et al. 2002; Alger 2012; Koch et al. 2014).

To date, DTI studies in OCD have implicated nearly all major WM tracts, including the cingulum bundle and the anterior and posterior limbs of the internal capsule, which are involved in the cortical‐striatal‐thalamic‐cortical loop; the corona radiata and the superior and inferior longitudinal fasciculus, which connect frontal to temporal‐parietal‐occipital cortices; and the bundle of corpus callosum, which subserves interhemispheric connectivity (Piras et al. 2013; Koch et al. 2014; Radua et al. 2014; Eng et al. 2015). However, the literature remains inconsistent with regard to the direction of effects, with some studies reporting significant FA decreases in OCD (Szeszko et al. 2005; Saito et al. 2008; Ha et al. 2009; Garibotto et al. 2010; Chiu et al. 2011; Admon et al. 2012; Oh et al. 2012) and others reporting increases in the same tracts (Cannistraro et al. 2007; Yoo et al. 2007; Nakamae et al. 2008; Li et al. 2011). Some of this variation may be attributable to the use of region of interest approaches (Cannistraro et al. 2007; Saito et al. 2008; Chiu et al. 2011; Lochner et al. 2012; Oh et al. 2012; Lopez et al. 2013), which are hypothesis driven but neglect other regions that are potentially affected (Koch et al. 2014). Other investigations have utilized whole‐brain voxel‐based morphometry (VBM) analysis (Szeszko et al. 2005; Yoo et al. 2007; Menzies et al. 2008b; Nakamae et al. 2008; Ha et al. 2009; Garibotto et al. 2010; Li et al. 2011; Admon et al. 2012), which suffers from several methodological limitations when applied to DTI data, particularly misregistration and partial volume effects (Jones et al. 2005). More recently, tract‐based spatial statistics (TBSS) have been advanced as a means of overcoming several of these limitations by performing whole‐brain voxel‐wise analysis to provide a more comprehensive measure of WM alterations (Smith et al. 2006; Koch et al. 2014). However, the studies that have examined adult OCD patients using whole‐brain TBSS so far do not paint a coherent picture (Bora et al. 2011; Fontenelle et al. 2011; Nakamae et al. 2011; Benedetti et al. 2013; Spalletta et al. 2014).

In addition to structural abnormalities, OCD patients show cognitive impairment in executive functions, attention, visuospatial abilities, and nonverbal memory (Nakao et al. 2014; Eng et al. 2015), many of which correlate with gray‐matter changes in frontal cortex, basal ganglia, and thalamus (Eng et al. 2015). However, while correlations between cognition and WM microstructural alterations have been found in healthy subjects (Burzynska et al. 2011) and patients with schizophrenia and bipolar disorder (Poletti et al. 2015; Roalf et al. 2015), this relationship has not been the focus of much investigation in OCD. To our knowledge, only one study has examined WM changes related to cognitive dysfunction in OCD, identifying a correlation between semantic fluency and FA in the corona radiata and corticospinal tract (Spalletta et al. 2014). However, these regions were not different between OCD and HC groups as a whole; as such, the meaning of this relationship is unclear. Nevertheless, this type of approach is critically important for understanding the mechanisms by which microstructural alterations impact behavior and may help explain some of the discrepancies in the literature.

This study used TBSS to investigate whole‐brain WM microstructure, indexed by FA, MD, and RD in patients with OCD compared with healthy controls (HC). We also explored the relationship of WM alterations with clinical and cognitive variables in OCD in order to investigate the structural correlates of behaviorally relevant features of the disorder.

## Materials and Methods

### Subjects and assessments

Subjects were recruited from psychiatric clinics at the Icahn School of Medicine at Mount Sinai (ISMMS) and from the greater New York metropolitan area. All subjects provided written informed consent, and the research was approved by the Institutional Review Board of the ISMMS. A total of 34 subjects were included in the analysis, comprising 16 patients with OCD and 18 HC. All patients met DSM‐IV criteria for current OCD, excluding primary hoarding subtypes (A.P.A. 1994). Exclusionary comorbid diagnoses for OCD patients included lifetime history of bipolar disorder or psychosis, as well as current posttraumatic stress disorder, panic disorder, or eating disorders. Axis I comorbidities consisted of generalized anxiety disorder (*n* = 4), trichotillomania (*n* = 1), excoriation (i.e., skin picking) disorder (*n* = 3), body dysmorphic disorder (*n* = 2), social phobia (*n* = 1), and major depressive disorder (*n* = 1).

To accurately reflect the severity range seen in typical OCD outpatients, subjects were not excluded on the basis of psychotropic medication status. Three patients were unmedicated at the time of the study; the remaining patients were taking selective serotonin reuptake inhibitors (SRIs), specifically fluoxetine (*n* = 4), sertraline (*n* = 4), escitalopram (*n* = 3), citalopram (*n* = 2), or venlafaxine (*n* = 1). Of these, three patients were taking additional adjunctive medications (bupropion and lamotrigine, *n* = 1; risperidone, *n* = 1; or aripiprazole, *n* = 1). Four patients also reported taking benzodiazepines as needed, but all refrained from doing so on the day of the MRI scan. HCs were free of any past or present psychiatric or neurological diagnoses as well as psychotropic medications.

All subjects were evaluated for DSM‐IV diagnoses using the Mini International Neuropsychiatric Interview (Sheehan et al. 1998). Symptoms of general anxiety and depression were quantified for both groups using Beck Anxiety Inventory and Beck Depression Inventory, respectively (Beck and Steer 1984; Beck et al. 1988). Obsessive–compulsive symptom severity was measured in the OCD group using the Yale‐Brown Obsessive–Compulsive Scale (Y‐BOCS) (Goodman et al. 1989).

Cognitive performance was assessed using a target detection task performed in the MRI scanner. The task consisted of 48 blocks of target detection. Each block was 15 s in duration and presented subjects with 15 sequential letters (750 ms per letter followed by a crosshair for 250 ms until the next letter appeared). Subjects were required to press one button for the target letter “a” (~30% of letters) and another button for all other letters. Prior to each target detection block, subjects performed one of four different tasks, each for 15 s on average: a color‐word Stroop conflict task, a positive event imagination task, a negative event imagination task, or rest. The imagination tasks involved thinking about personally relevant life events that the subject was looking forward to/wanted to happen (positive event imagination) or was not looking forward to/did not want to happen (negative event imagination). Target detection blocks were identical regardless of the preceding activity. Cognitive outcome consisted of variables percent commission errors (i.e., incorrect responses) and mean reaction time (RT) for correct responses. Both percent commission errors and mean RT were further broken down based on the preceding activity (i.e., percent commission errors and mean RT following the Stroop task, positive event imagination, negative event imagination, and resting condition). This task, while not a part of any standard neuropsychological battery of cognition, was designed to probe behavior associated with switching from a negative internal focus (i.e., imagining negative future events) to a nonaffective, externally oriented task (i.e., target detection). OCD is characterized by a difficulty in disengaging from imagined scenarios of harm or bad events, and this task was developed in attempts to capture that difficulty experimentally. Further details of this paradigm are described elsewhere (Stern et al. 2014).

Obsessive–compulsive disorder patients did not differ significantly from HC in age, years of education, or gender. As the majority of OCD patients included in our sample were taking SRI medication, we computed a fluoxetine equivalence dosage for each subject to examine any potential relationships between medication and DTI measures. Demographics, clinical information, and cognitive performance for the groups are shown in Tables 1 and 2.

**Table 1:** Demographic and Clinical Information

	OCD	HC
Sample size	16	18
Age (years)	27.6 (±6.8)	28.2 (±7.2)
Number of females (%)	11 (68.8%)	10 (55.6%)
Duration of illness (years)	15.1 (±6.7)	–
Y‐BOCS	19.7 (±5.75)	–
BAI	22.2 (±12.8)	3.0 (±6.1)
BDI	11.8 (±7.8)	1.4 (±1.8)

OCD, obsessive–compulsive disorder; HC, healthy controls; Y‐BOCS, Yale‐Brown obsessive–compulsive scale; BAI, Beck anxiety inventory; BDI, Beck depression inventory.

For all measures except sample size and number of females, group means are given with standard deviations in parentheses.

**Table 2:** Cognitive parameters in target detection task

	OCD	HC	*t*	*P*
Total errors during TD (percentage)	2.8 (±1.8)	1.8 (±1.2)	2.027	0.051^(^ * ^)^
Errors following Stroop	2.6 (±2.1)	2.4 (±1.7)	0.306	0.761
Errors following rest	3.2 (±2.3)	1.6 (±1.4)	2.321	0.029*
Errors following positive imagination	2.6 (±2.0)	1.8 (±1.8)	1.176	0.248
Errors following negative imagination	2.9 (±1.7)	1.1 (±1.1)	3.763	**0.001** *
Total mean RT of correct responses during TD	521.5 (±38.0)	510.0 (±34.5)	0.928	0.361
Mean RT following Stroop	525.4 (±37.1)	509.3 (±34.7)	1.303	0.202
Mean RT following rest	519.5 (±39.1)	506.6 (±33.0)	1.046	0.304
Mean RT following positive imagination	518.0 (±35.4)	511.8 (±38.6)	0.486	0.630
Mean RT following negative imagination	523.3 (±43.9)	512.1 (±34.5)	0.829	0.413

OCD, obsessive–compulsive disorder; HC, healthy controls; TD, target detection task; RT, reaction time. Errors refer to commission errors (wrong response selected).

^(*)^ *P* with marginal significance; *P <* *0.05 (uncorrected); *(bold) *P *<* *0.05 (corrected).

### MRI data acquisition and analysis

Diffusion‐weighted MRI scans were acquired on a 3T Phillips Allegra scanner using a 16‐channel head coil (42 gradient directions interleaved with 7 B_0_‐weighted volumes, 51 slices per volume). Data were preprocessed and analyzed using FSL 5.0 (Oxford University Centre FMRIB Software Library, http://www.fmrib.ox.ac.uk/fsl/) (Woolrich et al. 2009). First, the b0 images of each subject were skull‐stripped using the brain extraction tool. Data were then corrected for subject motion and eddy current‐induced geometrical distortions, and the diffusion sensitizing gradients (“bvecs”) were rotated to correct for motion. Next, using the FMRIB's Diffusion Toolbox (FDT), the diffusion tensor (DT) was estimated for each voxel via linear regression to derive FA, MD, and RD maps.

Subsequently, the TBSS package was used to perform voxel‐wise analyses of whole‐brain WM measures (http://www.fmrib.ox.ac.uk/fsl/tbss/index.html) (Woolrich et al. 2009). Briefly, individual FA images underwent nonlinear registration to the FMRIB58_FA template space and were averaged to create a mean FA image. This was then thinned to create a WM tract “skeleton” using the default FA threshold of 0.2 to exclude non‐WM voxels. Each subject's aligned FA map was then projected onto this skeleton, resulting in an alignment‐invariant representation of the central trajectory of WM pathways for all subjects. This process was repeated for each subject's MD and RD map using the individual registration and projection vectors obtained in the FA nonlinear registration and skeletonization.

Voxel‐wise differences in FA, MD, and RD values between OCD patients and HC were tested using permutation‐based inference for nonparametric statistical thresholding (FSL's “randomize” function) (Nichols and Holmes 2002) and two‐sample t‐tests. The number of permutations was set to 5000 to allow robust statistical inference. Age and sex were entered into the analysis as confound regressors. For between‐group comparisons, a family‐wise error corrected threshold of *P *<* *0.05 was selected using the randomize tool's threshold‐free cluster enhancement (TFCE) option (Smith and Nichols 2009). The WM tracts were identified using the ICBM‐DTI‐81 white‐matter labels atlas included with FSL (Wakana et al. 2007; Hua et al. 2008). For correlation analyses, skeletonized voxels with significantly different FA, MD, or RD values between patients and controls were inverse transformed back into each subject's native space and individual values were extracted.

### Behavioral group comparison and correlation analyses

*T*‐tests were performed to compare cognitive performance between OCD patients and HC on all outcome variables. Correlation analyses were conducted for those DTI parameters that differed significantly between OCD and HC groups. Within the entire sample, partial correlations controlling for group status were performed between DTI parameters and (1) the cognitive variable showing a group difference at the corrected significance threshold (i.e., errors during target detection following negative event imagination, see Results below) and (2) clinical variables obtained for both groups (i.e., BDI and BAI). The same correlation analyses were subsequently repeated within each group. For the OCD group, further correlation analyses were performed between DTI parameters and diagnosis‐specific clinical variables (i.e., Y‐BOCS, medication dosage, and illness duration). As Shapiro–Wilks testing indicated a nonnormal distribution for cognitive and clinical variables, Spearman correlations were employed for intragroup testing. All correlation results were considered significant at the Bonferroni‐corrected *P *<* *0.05 level.

## Results

### Comparison of DTI metrics between OCD and HC

**Figure 1:**
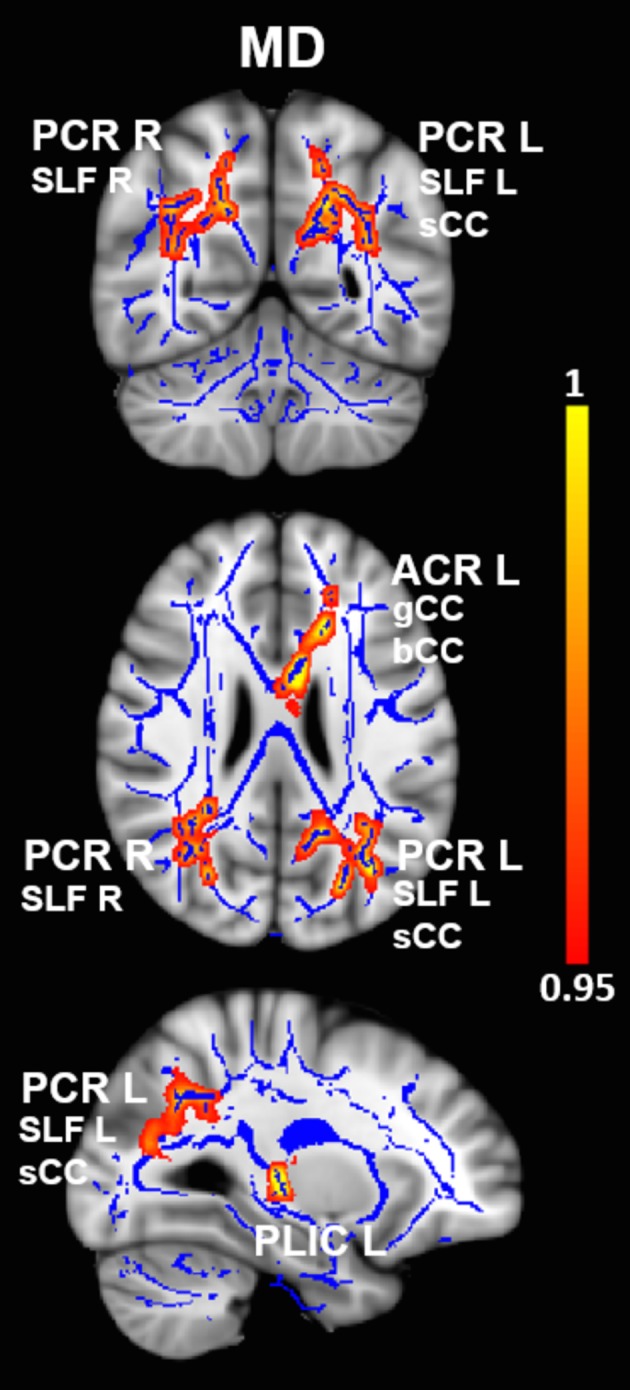
Differences in mean diffusivity values in OCD vs. HC. Results from between‐group comparison showing clusters with significantly increased MD values in OCD patients compared with HC (*P*_*TFCE*_ < 0.05, *red‐yellow*). The group‐averaged white‐matter skeleton (FA threshold > 0.2) is shown in *blue*. Group differences are mapped onto a standard T1 Montreal Neurological Institute (MNI) template. Images are in radiological convention (i.e., findings in the left hemisphere are displayed on the right and *vice versa*). The color bar represents statistical significance (1 minus *P*‐value). MD, mean diffusivity; OCD, obsessive–compulsive disorder; HC, healthy controls; PCR L/R, left and right posterior corona radiata; SLF L/R, left and right superior longitudinal fasciculus; ACR L, left anterior corona radiata; sCC, splenium of the corpus callosum; gCC, genu of the corpus callosum; bCC, body of the corpus callosum; PLIC L, left posterior limb of the internal capsule.

**Figure 2:**
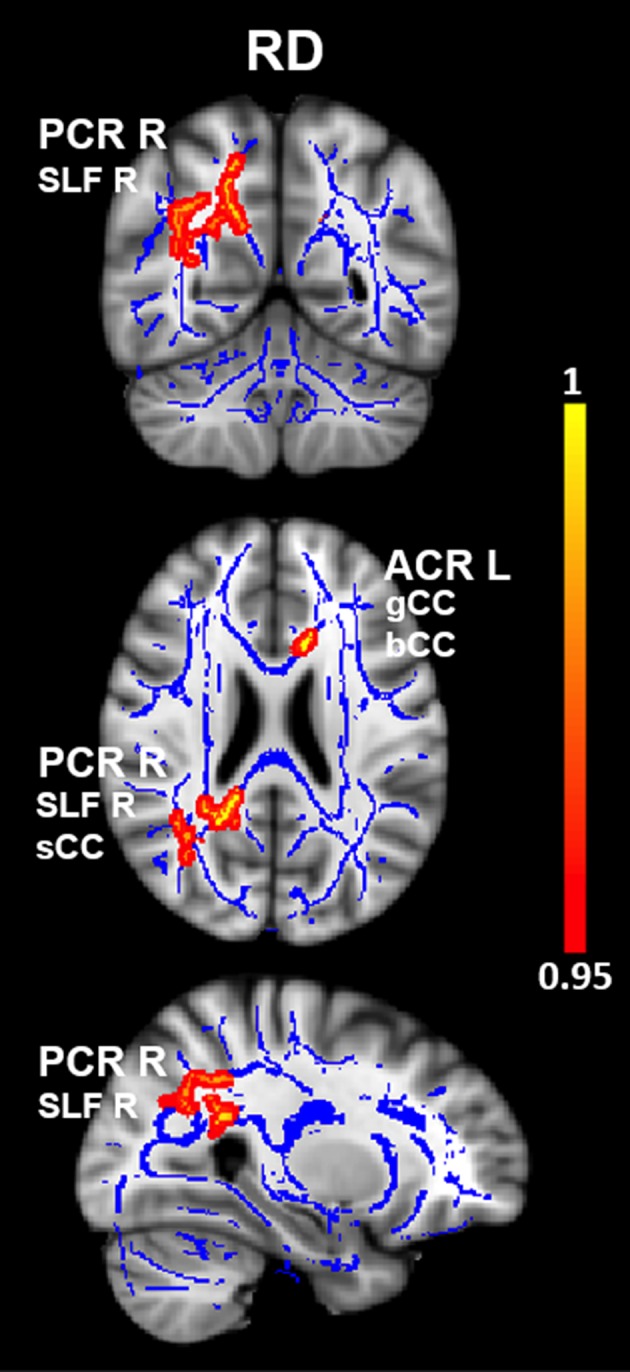
Differences in radial diffusivity values in OCD vs. HC. Results from between‐group comparison showing clusters with significantly increased RD values in OCD patients compared with HC (*P*_*TFCE*_ < 0.05, *red‐yellow*). The group‐averaged white‐matter skeleton (FA threshold > 0.2) is represented in *blue*. Group differences are mapped onto a standard T1 Montreal Neurological Institute (MNI) template. Images are in radiological convention (i.e., findings in the left hemisphere are displayed on the right and *vice versa*). The color bar represents statistical significance (1 minus *P*‐value). RD, radial diffusivity; OCD, obsessive–compulsive disorder; HC, healthy controls; PCR R, right posterior corona radiata; SLF R, right superior longitudinal fasciculus; ACR L, left anterior corona radiata; sCC, splenium of the corpus callosum; gCC, genu of the corpus callosum; bCC, body of the corpus callosum.

Mean diffusivity values were significantly increased in OCD patients compared with HC in four clusters. The first cluster mainly encompassed the left posterior corona radiata (PCR L) near the temporoparietal junction and also included parts of the left superior longitudinal fasciculus (SLF L) and splenium of corpus callosum (sCC). The second cluster included the right posterior corona radiata (PCR R) near the temporoparietal junction, as well as the right superior longitudinal fasciculus (SLF R). The third cluster included the left anterior corona radiata (ACR L) near the anterior cingulate cortex, as well as the genu and body of corpus callosum (gCC, bCC). Finally, the fourth cluster included the left posterior limb of the internal capsule (PLIC L). See Figure 1 and Table 3 for more details. Interestingly, RD values were also significantly increased in OCD patients compared with HC in two of the same clusters, one which included the PCR R, SLF R, and sCC, and the other which encompassed the ACR L, gCC, and bCC (Fig. 2 and Table 4). No significant differences in FA were detected between groups at the *P*_*TFCE*_ < 0.05 level.

**Table 3:** White‐matter clusters showing increased mean diffusivity in OCD

WM clusters	MNI coordinates			CS	Corrected *P*
	*x*	*y*	*z*		
PCR L	−32	−61	28	1923	0.02
SLF L					
sCC					
PCR R	34	−61	26	1361	0.013
SFL R					
ACR L	−17	21	30	789	0.042
gCC					
bCC					
PLIC L	−28	−22	1	147	0.042

OCD, obsessive–compulsive disorder; MD, mean diffusivity; CS, cluster size (i.e., number of voxels); PCR L, left posterior corona radiata; PCR R, right posterior corona radiata; ACR L, left anterior corona radiata, PLIC L, left posterior limb of the internal capsule; SLF L, left superior longitudinal fasciculus; SLF R, right superior longitudinal fasciculus; sCC, splenium of corpus callosum; gCC, genu of corpus callosum; bCC, body of corpus callosum.

White‐matter clusters with significantly increased MD (*P*_TFCE_ < 0.05) in OCD vs. HC subjects. Coordinates indicate the location of the cluster peak in Montreal Neurological Institute (MNI) convention.

**Table 4:** White‐matter clusters showing increased radial diffusivity in OCD

WM clusters	MNI coordinates			CS	Corrected *P*
	*x*	*y*	*z*		
PCR R	24	−65	32	1795	0.021
SFL R					
sCC					
ACR L	−11	22	19	106	0.047
gCC					
bCC					

OCD, obsessive–compulsive disorder; RD, radial diffusivity; CS, cluster size (i.e., number of voxels); PCR R, right posterior corona radiata; ACR L, left anterior corona radiate; SLF R, right superior longitudinal fasciculus; sCC, splenium of corpus callosum; gCC, genu of corpus callosum; bCC, body of corpus callosum.

White‐matter clusters with significantly increased RD (*P*_TFCE_ < 0.05) in OCD vs. HC subjects. Coordinates indicate the location of the cluster peak in Montreal Neurological Institute (MNI) convention.

### Cognitive performance and correlations with DTI metrics

In the cognitive task, OCD patients made significantly more commission errors during target detection following negative event imagination (*t* = 3.763; *P *=* *0.001) and following rest (*t* = 2.321; *P *=* *0.029) than HC. These findings also drove a marginally significant increase in total percent commission errors (*t* = 2.027; *P *=* *0.051). No significant differences were found in the other cognitive variables. Only the group difference in commission errors during target detection following negative event imagination survived Bonferroni correction (Table 2).

**Figure 3:**
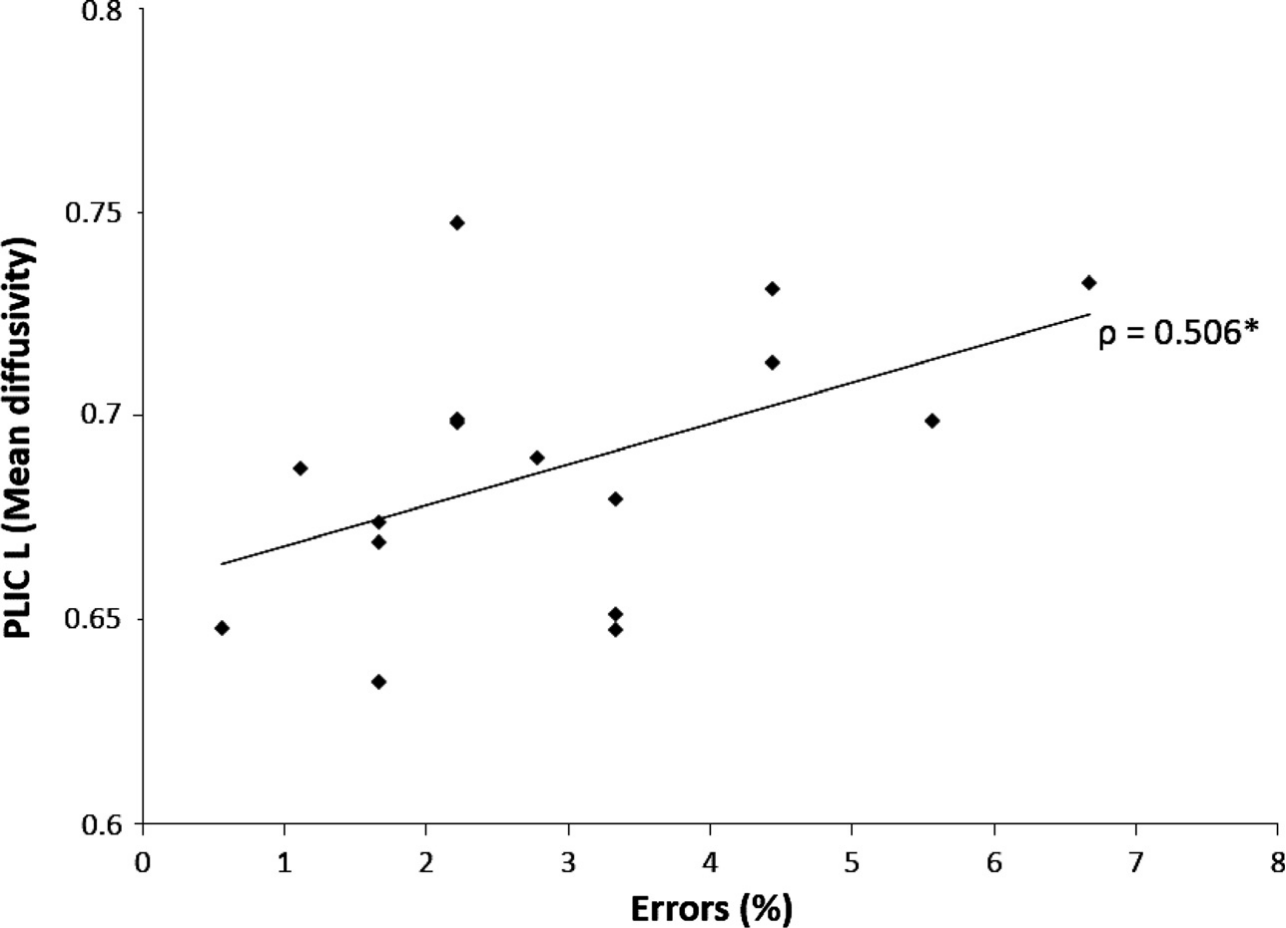
Correlations between cognitive performance and mean diffusivity. Positive correlation between mean diffusivity in the PLIC L cluster and percent commission errors following negative event imagination within the OCD group; *P *=* *0.045. PLIC L, left posterior limb of the internal capsule.

We next performed a partial correlation analysis, controlling for group status, between the cognitive variable showing group differences (i.e., commission errors following negative event imagination) and MD values from the clusters showing group differences in MD. This revealed a positive correlation between percent errors during target detection following negative event imagination and MD in the PLIC L cluster (r = 0.505; *P *=* *0.003). This correlation was significant within the OCD group (*ρ *= 0.506; *P *=* *0.045) (Fig. 3), but not in the HC group (*ρ *= 0.398; *P *=* *0.102). We did not find any significant correlations between commission errors following negative event imagination and MD values for the other clusters where MD differed between the groups. There were no significant correlations between MD values and BAI or BDI either across the entire sample or within groups. Similarly, there were no significant correlations between Y‐BOCS scores or illness duration and MD values within the OCD group. However, medication dosage correlated positively with MD in the PCR L (*ρ *= 0.564; *P *=* *0.023) and ACR L (*ρ *= 0.542, *P *=* *0.030) clusters, but not in the PCR R or PLIC L. We did not find any significant correlations between RD values in the two significant RD clusters and any of the cognitive or clinical variables.

## Discussion

In this study, we used TBSS to analyze whole‐brain WM microstructure in OCD, finding increased mean diffusivity in several regions including the posterior and anterior corona radiata, superior longitudinal fasciculus, corpus callosum, and posterior limb of the internal capsule. Radial diffusivity was also increased in some of the same tracts (right posterior corona radiata, right superior longitudinal fasciculus, left anterior corona radiata, and corpus callosum), suggesting disrupted myelination in these regions (Song et al. 2002; Alger 2012). Furthermore, increased MD in the internal capsule was correlated with the percentage of errors made during a target detection task following negative event imagination, which itself was significantly greater in the OCD group.

To date, a minority of studies investigating WM alterations in adult OCD patients have utilized the whole‐brain TBSS approach (Bora et al. 2011; Fontenelle et al. 2011; Nakamae et al. 2011; Benedetti et al. 2013; Koch et al. 2014; Spalletta et al. 2014). Our findings are consistent with these previous TBSS studies, identifying abnormalities in the corona radiata, internal capsule, corpus callosum, and superior longitudinal fasciculus (Bora et al. 2011; Fontenelle et al. 2011; Nakamae et al. 2011; Benedetti et al. 2013; Spalletta et al. 2014). Behaviorally, OCD patients made significantly more errors during a target detection task compared with HC, an effect that was particularly robust when they had previously been imagining personal negative events that could happen to them. This behavioral finding is especially relevant for understanding cognitive impairments in the disorder and supports the notion that OCD patients may be unable to disengage from negative internal thoughts in order to efficiently perform external cognitive tasks such as target detection (Stern and Taylor 2014; Stern et al. 2014). Critically, within the OCD group, this cognitive deficit was associated with increased mean diffusivity in the posterior limb of the internal capsule. The finding is particularly interesting in light of recent studies indicating that deep brain stimulation of the anterior limb of the internal capsule is efficacious for treating refractory OCD. The internal capsule is situated between basal ganglia (the caudate and lentiform nucleus) and thalamus regions and consists of both ascending and descending projection fibers. Its anterior limb mainly contains the anterior thalamic radiation connecting the thalamus to frontal lobe and is involved in fronto‐striato‐thalamo‐cortical loops implicated in OCD (Menzies et al. 2008a; Piras et al. 2013; Cleary et al. 2015). The anterior part of the posterior limb contains fibers of the corticospinal tract, while the posterior part of the posterior limb (the region identified in our analyses) conveys the superior thalamic radiations to sensorimotor cortical areas, as well as the posterior thalamic radiation to occipital, temporal, and parietal lobes (Wakana et al. 2004; Kim et al. 2008). Thus, this work extends prior findings of altered WM in fronto‐striato‐thalamo‐cortical loops in OCD (Menzies et al. 2008a; Piras et al. 2013; Cleary et al. 2015) to include a wider array of anatomical connections.

The exact mechanism linking WM alterations with specific cognitive disturbances in OCD remains unclear. In our sample, OCD patients showed abnormalities in several regions associated with large‐scale networks in the brain (van den Heuvel et al. 2009). In particular, the superior longitudinal fasciculus connects regions of the frontoparietal “control” network, while the genu of the corpus callosum has been shown to connect regions of the “default mode network” (DMN) (van den Heuvel et al. 2009). Furthermore, the splenium of the corpus callosum is immediately rostral to retrosplenial/posterior cingulate cortex (PCC), a key DMN hub (Buckner et al. 2008). Indeed, previous works indicated that greater MD in the splenium of the corpus callosum is correlated with functional connectivity of the PCC (Sharp et al. 2011). As such, it is tempting to propose that the abnormalities we report may be associated with altered large‐scale network functioning in OCD. In particular, the DMN is implicated in internally focused future thought and imagination (Buckner et al. 2008), and previous work by our group and others have reported abnormal functioning of DMN regions during fMRI tasks in OCD (Fitzgerald et al. 2005; Stern et al. 2011, 2012, 2013; Harrison et al. 2012; Milad et al. 2013). Somewhat surprisingly, diffusivity in these clusters was not correlated with behavior in the task; instead, we identified a relationship between white‐matter alterations in the posterior limb of the internal capsule and errors during target detection following negative internal thoughts, suggesting that white‐matter microstructure in this region may be linked to OCD‐related behaviors. It is unclear why we did not find a relationship between task‐related errors and MD in the other regions that differed significantly between OCD and HC groups (particularly regions related to DMN functioning), although this may be due to the fact that these clusters were larger and included multiple tracts.

Finally, we found no relationships between levels of OCD severity, depression, or anxiety and MD or RD in any of the areas that differed between groups. This suggests that WM abnormalities may be more closely related to intermediate behavioral dysfunction or “endophenotypic” differences than to general measures of symptom severity. Indeed, prior TBSS studies have also not found relationships between WM microstructure and symptom severity (Bora et al. 2011; Nakamae et al. 2011), although one group reported clinical correlations between Y‐BOCS total score and FA and MD values in corpus callosum (Fontenelle et al. 2011).

A number of limitations to this study should be noted. These included the relatively small sample size; although we were able to detect significant differences between OCD patients and HC at stringent thresholds even with these smaller numbers, it will be important to replicate these findings in a large sample. Another potential caveat is that most of the OCD patients included in our sample were medicated. While SRI medication is typical among OCD patients, group differences in two clusters (centered on posterior and anterior corona radiata in the left hemisphere) did correlate with dosage, suggesting that certain DTI abnormalities may be associated with pharmacotherapy in a dose‐dependent manner. Although few previous studies have investigated the relationship between medication and WM microstructure, at least one study did find that diffusivity differences between OCD and HC groups (including in the left corona radiata but not the internal capsule) appeared to be driven by the medicated patients (Benedetti et al. 2013). However, other studies have detected DTI alterations (including in the corpus callosum and internal capsule) in unmedicated patients (Bora et al. 2011; Nakamae et al. 2011) or a possible partial reversion of WM changes by SRI treatment (Fan et al. 2012). Therefore, the role of medication treatment on WM microstructure in OCD is still an open question, although it appears likely that SRIs are capable of altering myelin through effects on neuroglia signaling and other mechanisms (Bartzokis 2012; Benedetti et al. 2013). While our current sample was underpowered to examine medicated and unmedicated patients separately, future work will aim to determine whether unmedicated patients show similar MD and RD abnormalities as those observed here. It is important to note that MD values in the posterior limb of the internal capsule (and posterior corona radiata on the right side) were not related to SRI dosage, suggesting that the relationship identified between cognitive disturbance and internal capsule WM alterations is not due to medication effects. Finally, although a tractography approach would have been of interest to delineate the fibers passing trough significant clusters, our DTI acquisition was not optimized for this analysis. Future studies with larger samples of patients and more advanced DTI acquisition schemes are warranted to better characterize WM abnormalities in OCD.

In conclusion, our findings indicated that OCD patients showed WM alterations mainly in posterior and anterior corona radiata, corpus callosum, and posterior limb of the internal capsule. In particular, diffusivity in the posterior limb of the internal capsule correlated with behavioral difficulties that may be related to impaired disengagement from negative internal thoughts, suggesting that microstructural WM changes may have functional consequences for the disorder. If confirmed and extended, these results may improve understanding of the pathophysiology of OCD.

## Conflict of interests

All authors have no conflict of interest to declare.
